# Co-operative transitions of responsive-polymer coated gold nanoparticles; precision tuning and direct evidence for co-operative aggregation†
†Electronic supplementary information (ESI) available. See DOI: 10.1039/c6tb01336h



**DOI:** 10.1039/c6tb01336h

**Published:** 2016-08-02

**Authors:** Sangho Won, Daniel J. Phillips, Marc Walker, Matthew I. Gibson

**Affiliations:** a Department of Chemistry , University of Warwick , Coventry , CV4 7AL , UK . Email: M.I.Gibson@warwick.ac.uk; b Department of Physics , University of Warwick , Coventry , CV4 7AL , UK; c Warwick Medical School , University of Warwick , Coventry , CV4 7AL , UK

## Abstract

Responsive polymers and polymer-coated nanoparticles have many potential bio-applications with the crucial parameter being the exact temperature where the transition occurs. Chemical modification of hydrophobic/hydrophilic or ligand binding sites has been widely explored as a tool for controlling this transition, but requires the synthesis of many different components to achieve precise control. This study reports an extensive investigation into the use of blending (*i.e.* mixing) as a powerful tool to modulate the transition temperature of poly(*N*-isopropylacrylamide) (PNIPAM) coated gold nanoparticles. By simply mixing two nanoparticles of different compositions, precise control over the transition temperature can be imposed. This was shown to be flexible to all possible mixing parameters (different polymers on different particles, different polymers on same particles and different sized particles with identical/different polymers). Evidence of the co-operative aggregation of differently sized nanoparticles (with different cloud points) is shown using transmission electron microscopy; particles with higher cloud points aggregate with those with lower cloud points with homo-aggregates not seen, demonstrating the co-operative behaviour. These interactions, and the opportunities for transition tuning will have implications in the rational design of responsive biomaterials.

## Abstract 2

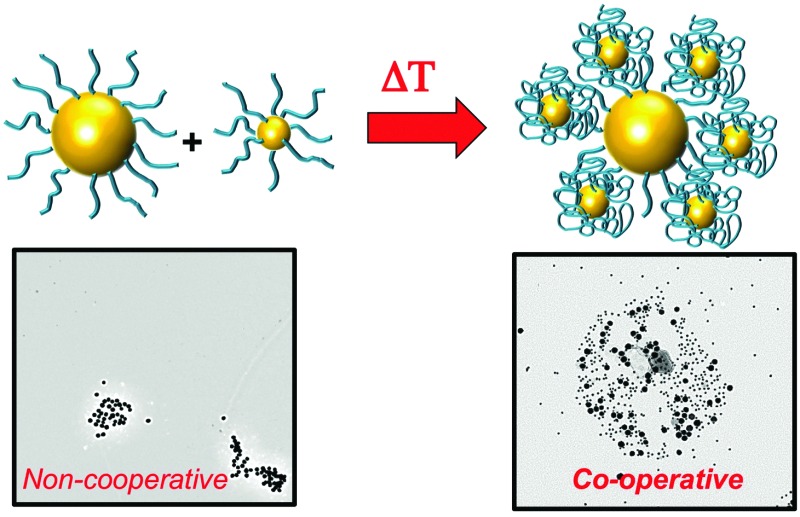
Responsive polymers and polymer-coated nanoparticles have many potential bio-applications with the crucial parameter being the exact temperature where the transition occurs.

## Introduction

Stimuli-responsive polymers can change their conformation and hence properties when external stimuli, such as temperature,^1^ pH,^2^ light,^3^ ions,^4^ electric,^5^ magnetic field^6^ and biochemical agents^7^ are applied. These unique properties and abilities have been formulated with polymers for various biomedical fields in a number of forms including simple polymer solutions,^8^ hydrogels,^9^ micelles,^10^ films and layers.^11^ Especially, thermo-responsive polymers are the most commonly used for both *in vitro* ^12^ and *in vivo* ^13^ biomedical applications. Thermo-responsive polymers in solution display a lower critical solution temperature (LCST) or upper critical solution temperature (UCST). The LCST is the point where the polymer phase separates such that the polymer solution is miscible below the critical temperature whereas polymer solution is immiscible (precipitates) above it.

Poly(*N*-isopropylacrylamide) (PNIPAM) is one of the most well-known temperature-responsive polymers.^14,15^ In general, the LCST transition of PNIPAM is observed to be between 30 °C to 45 °C.^16^ Several factors including molecular weight,^17^ terminal group of the polymer,^18^ polymer concentration,^19^ pH in solution and the presence of salt^20^ as well as self assembly^21^ have significant effects on the observed transition temperatures.

By employing RAFT (reversible addition fragmentation and transfer) polymerization, pNIPAM (and indeed many other classes of polymers) can be directly incorporated onto the surface of gold nanoparticles due to the quantitative installation of a (masked) thiol at each α-chain end. Responsive polymer coated gold nanoparticles retain sensitivity to external stimuli^22^ and can be applied in wide range of therapeutic applications including a catalysis, diagnosis, imaging and photoelectronic device.^23,24^ It has been reported that PNIPAM coated gold nanoparticles can be used to drive cellular uptake upon increasing the temperature above the solution temperature due to a shift in the lipophilicity of the particle promoting membrane interactions^24a,25^ and is an attract route to gain entry into cells for delivery applications. However, to achieve this (or other) goals, precise control over the transition temperature is essential. It has been reported by Fernández-Trillo *et al.* that the cloud point of elastin-mimetic polymers could be tuned by simple mixing polymers with different cloud points.^26^ This has been shown to be the case for some polymers but also polymer-grafted nanoparticles.^27^ However, other classes of polymers failed to show any co-operativity with distinct ‘steps’ seen in mixtures of polymers such as elastin peptides or poly(oligo[ethyleneglycol methacrylates]) implying this is a unique property.^28^

The above raises questions about the scope of the blending approach but also the mechanism – for a single transition to occur, the particle/polymer with the higher cloud point must preferentially interact with the particle/polymer with a lower cloud point (otherwise it would not aggregate at temperatures below its own transition temperature). This implies a more complex inter-relationship between the entities than is often considered, and thus far there is no direct evidence of these interactions, as only turbidimetry is employed.

In this manuscript, the co-operative aggregation of pNIPAM coated nanoparticles is studied in detail and the limits and scope of the blending (using all possible mixing parameters of particles and polymers) is studied using a range of techniques. Crucially, the size-dependant co-operative transitions are studied using electron microscopy, enabling the identity of each interacting component to be assigned to direct observe the co-operativity for the first time.

## Results and discussion

**Fig. 1:**
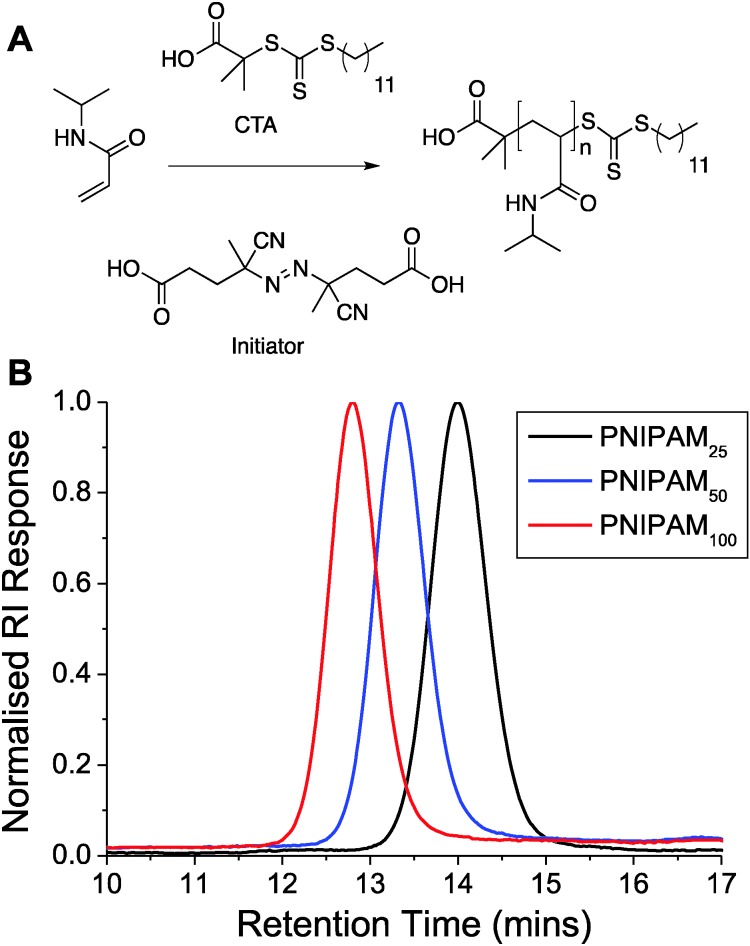
(A) Synthetic route to pNIPAMs; (B) SEC analysis of polymers.

To access the range of well-defined PNIPAM's required for this study RAFT (reversible addition fragmentation chain transfer) polymerization was employed, Fig. 1A. A trithiocarbonate RAFT agent was chosen, and synthesized, based on its reported utility for control over the RAFT polymerization, which also inserts the desired thiol end-group for later immobilization onto gold nanoparticles. NIPAM was polymerized in dioxane and following isolation by precipitation was characterized by ^1^H and ^13^C NMR and SEC (size exclusion chromatography). SEC confirmed a controlled polymerization with narrow dispersity values and the observed molecular weights agreeing well with those predicted from the monomer : initiator feed ratio, Table 1 and Fig. 1. The polymers are labelled from here based on their targeted degree of polymerization; PNIPAM_25_, PNIPAM_50_ and PNIPAM_100_.

**Table 1:** Characterisation of polymers

Polymer	[M]/[CTA]/[I] [mol]	*M*_n Target_ [g mol^–1^]	Conversion^*a*^ [%]	*M*_n Theo_ ^*b*^ [g mol^–1^]	*M*_n SEC_ ^*c*^ [g mol^–1^]	*M*_w_/*M*_n_ [—]
PNIPAM_25_	25/1/0.2	2800	87	2500	2900	1.07
PNIPAM_50_	50/1/0.2	5700	84	4800	4900	1.07
PNIPAM_100_	100/1/0.2	11 300	72	8100	7700	1.10

^*a*^Determined ^1^H NMR.

^*b*^Calculated from the [monomer] : [CTA] ratio and of conversion.

^*c*^Determined by SEC in DMF using PMMA standards.

The polymers were then evaluated for their thermo-responsive behavior both in water and in saline, as this is crucial for the measurements later in this manuscript as ‘grafted to’ gold nanoparticle polymer hybrids require salt to screen their overall net negative charge (*vide infra*). UV-Vis spectroscopy was used to determine the CP (cloud point) of polymer defined as being the point of 50% transmittance and the results of this (at 2.5 mg mL^–1^) are shown in Table 2. (Note, the cloud point is distinct from, and is the macroscopic effect associated with, an LCST). In water the polymers all had CPs from 30–38 °C as expected, but in buffer an unexpectedly high cloud point was observed with the shortest polymer having a transition above 60 °C. We ascribed this to partial self assembly of the shortest polymers into micelles due to the hydrophobic end group, and hence the transitions are for nanostructures rather than the polymers, but is outside of the scope of this study (focused on the nanoparticles, *vide infra*). To probe any co-operative effects, a pair of polymers having different molecular weights below ∼10 kg mol^–1^ were tested as PNIPAM does not have a strong molecular weight dependence on its LCST above this value.^29^ PNIPAM_50_ (4.9 kDa) and PNIPAM_100_ (7.7 kDa) were prepared at 2.5 mg mL^–1^ in PBS solution. Table 2 also shows transition temperature for blends of PNIPAM_50_ and PNIPAM_100_ at various weight fraction compositions. There are two independent transition temperature of each pure polymer, PNIPAM_50_ has a transition temperature of 47 °C whereas PNIPAM_100_ has a transition temperature of 41 °C. Blends of these polymers at all weight fractions shows a clear transition between 41–47 °C. The CPs of polymer mixture were shifted towards higher as increasing amounts of PNIPAM_50_ to PNIPAM_100_ with a cloud point of 42 °C, 43 °C and 45 °C, respectively.

**Table 2:** Cloud points for each polymer and polymer mixture in different media

Polymer	Weight fraction [%]	Cloud point_water_ ^*a*^ [°C]	Cloud point_PBS_ ^*b*^ [°C]
PNIPAM_25_	100	33	61
PNIPAM_50_	100	36	47
PNIPAM_100_	100	38	41
PNIPAM_50_ : PNIPAM_100_	25 : 75	—	42
PNIPAM_50_ : PNIPAM_100_	50 : 50	—	43
PNIPAM_50_ : PNIPAM_100_	75 : 25	—	45

^*a*^pure water and

^*b*^PBS buffer upon heating from 25 °C to 85 °C, 2.5 mg mL^–1^ polymer concentration. CPs of PNIPAM mixture were not measured in water due to the very narrow gap between each polymers CP.

**Scheme 1:**
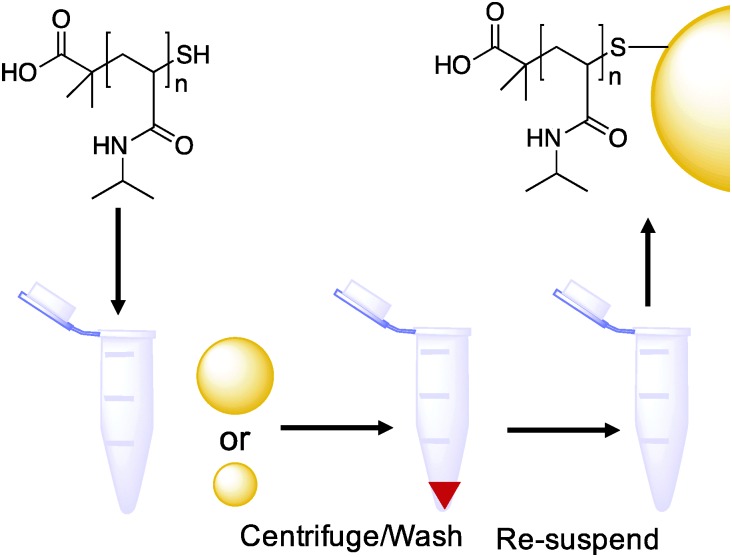
Synthesis of thermo-responsive polymer (PNIPAM) and polymer/gold hybrid nanoparticles.

With the function of the polymers and their co-operative nature confirmed, PNIPAM coated gold nanoparticles were synthesized as shown in Scheme 1. Briefly, the RAFTed polymers were mixed onto pre-made citrate stabilized gold nanoparticles by a simple mixture procedure, which we have used previously. Excess polymer was removed by repeated centrifugation samples to ensure only polymer-coated nanoparticles were investigated and not free polymer (Table 3).

**Table 3:** Characterization of the polymer coated nanoparticles

Particle^*a*^	SPR_max_ [nm]	*d*_DLS_ ^*b*^ [nm]	Cloud point^*c*^ [°C]
Bare Au 15 nm	520	18	—
PNIPAM_25_@Au_15_	524	24	—
PNIPAM_50_@Au_15_	524	32	74
PNIPAM_100_@Au_15_	525	37	55
Bare Au 40 nm	526	42	—
PNIPAM_25_@Au_40_	530	50	—
PNIPAM_50_@Au_40_	531	56	80
PNIPAM_100_@Au_40_	531	61	60

^*a*^X@Au_*m*_: X = polymer coating used; *m* = diameter of gold nanoparticle.

^*b*^ *d*_DLS_(nm): *Z*-average diameter as determined by DLS.

^*c*^Measured in PBS upon heating from 25 °C to 85 °C, 0.029 mg mL^–1^ total gold concentration.

**Fig. 2:**
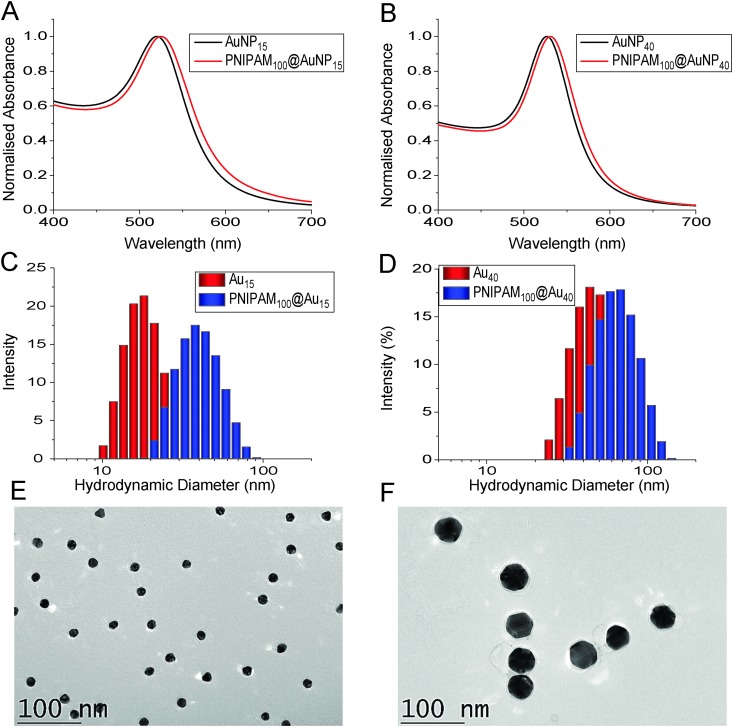
Size characterization of PNIPMA100@Au15 and NIPAM100@Au40 nanoparticles. (A + B) UV-Vis analysis before and after polymer coating; (C + D) DLS analysis before and after polymer coating; (E + F) TEM of polymer coated gold nanoparticles.

The nanoparticles were characterized by a range of techniques including TEM, DLS and XPS. The hydrodynamic diameter of uncoated gold nanoparticles was 18 nm and 42 nm by DLS measurements while after PNIPAM coating diameter were increased to 37 nm and 61 nm, respectively confirming the surface-tethering of the polymer chains. The surface plasmon resonance bands were also red shifted to longer wavelengths from 520 to 525 nm for 18 nm gold nanoparticles and 526 to 531 nm for 42 nm gold nanoparticles consistent with successful functionalisation. The particles were also imaged by TEM after functionalization with the polymers (the non-coated particles tended to aggregate on the TEM grid, see Fig. S6, ESI†). A uniform distribution of spherical core nanoparticles with an average particle size of 15.2 ± 1.4 nm and 40.7 ± 4.0 nm were observed in TEM and there was no evidence of agglomeration or ripening of the gold (Fig. 2).

**Fig. 3:**
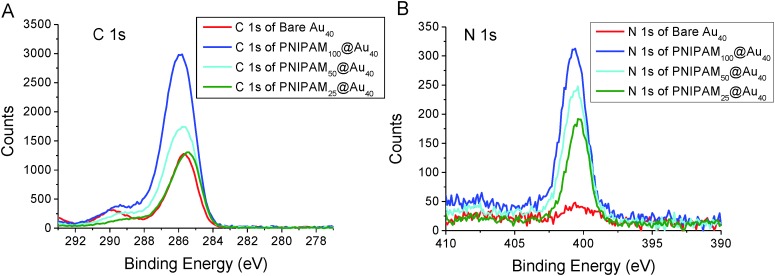
X-ray photoelectron spectroscopy analysis of PNIPAM functionalized gold nanoparticles. Representative high-resolution XPS spectrum of C1s and N1s region before and after various PNIPAM conjugation. (A) Carbon (C1s) peak and (B) nitrogen (N1s) peak from the XPS analysis of polymer/gold hybrid nanoparticles.

Additional characterisation was conducted using surface X-ray photoelectron spectroscopy (XPS) using gold particles deposited on silicon supports. As can be seen in Fig. 3, both carbon (C1s) and nitrogen (N1s) region (at around 399.7 eV) of the spectra for the PNIPAM coated gold nanoparticles is broader than the bare gold nanoparticles. This is due to the C–N peak at around 286.2 eV. The presence of the C=O peak at around 287.8 eV indicates clear evidence of the presence of PNIPAM on the gold surface. Also, the intensity of both carbon and nitrogen peak increased higher after conjugation of longer PNIPAM polymer chain because of the higher concentration of carbon and nitrogen are incorporated in longer PNIPAM chain.

**Fig. 4:**
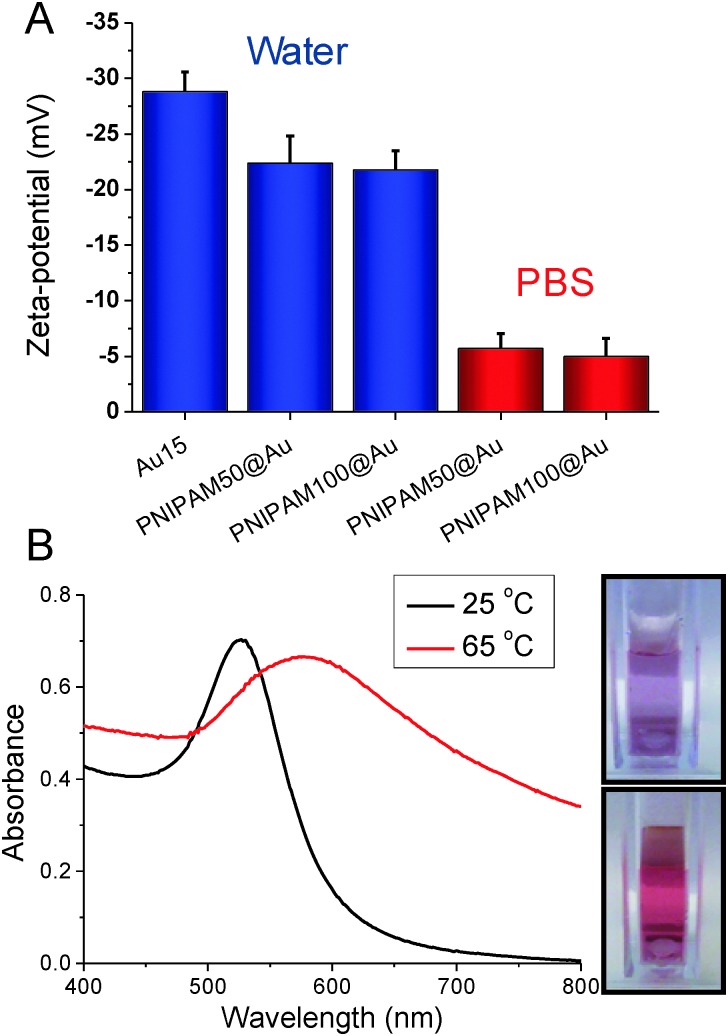
Solution properties of polymer coated nanoparticles. (A) Zeta-potential analysis of nanoparticles in water, and in phosphate buffered saline; (B) example UV-Vis spectra showing effect of heating above the transition temperature. Inset images show red-blue colour shift upon aggregation.

The PNIPAM@Au particles could now be tested for their responsive behaviour. As reported by Hoogenboom *et al.*,^30^ in water no transition was seen in the temperature range tested (25–85 °C), but when repeated in PBS a clear transition could be seen. These observations were attributed to the residual citrate on the nanoparticle surface, which was still negative charged (measured by zeta-potential) even after polymer coating, but approached neutrality in PBS. Fig. 4B shows the effect of heating the particles in PBS above their CP, with a clear red shift (long wavelength) in their absorption spectra, with an increase at 700 nm and red-blue colour shift. Aggregation was further confirmed by variable temperature DLS which showed an increase in hydrodynamic diameter above the transition temperature due to increased hydrophobicity of the particles (ESI†).

**Fig. 5:**
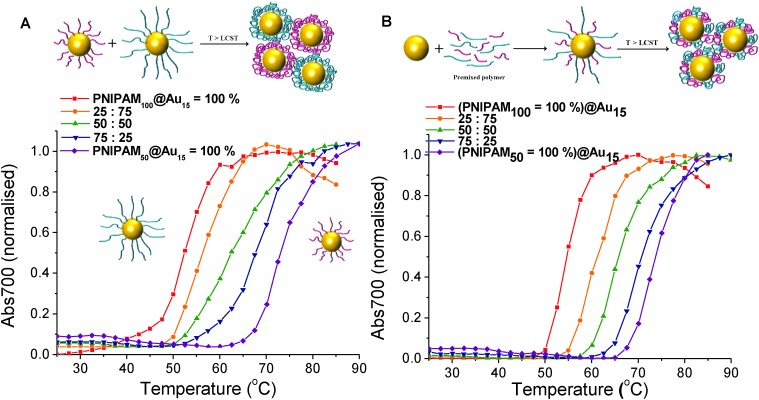
Turbidimetry scans (absorbance at 700 nm) of (A) PNIPAM_50_@Au_15_ and PNIPAM_100_@Au_15_ mixture of the particles with different mass fraction, (B) pre-mixture of PNIPAM_50_ and PNIPAM_100_ with different mass fraction coated Au_15_ in PBS solution. In all cases the total gold core concentration of the solutions was 0.029 mg mL^–1^.

The main aim of this study was to probe, and provide direct evidence for, the co-operative LCST transitions of polymer coated gold nanoparticles as a route to not only fine-tune their transition temperatures but also a way to enhance biosensing or generate complex assemblies of mixed particles. We have previously shown that pNIPAM homopolymers and various soft and hard nanoparticles show ‘co-operative LCST’ behaviour, but the scope and limitations of this property has not been fully investigated nor direct evidence for non-identical polymers or particles interacting.^27,31^ To probe the potential for mixing, we investigate here a range of different combinations of polymers and nanoparticles. Firstly, PNIPAM_50_ and PNIPAM_100_ were immobilised onto Au_15_. These nanoparticles solutions were then mixed with different weight ratio of 25 : 75, 50 : 50 and 75 : 25 respectively and their responsive transitions measured by UV-Vis, Fig. 5A. For the above mixtures, in each case a single transition temperature was observed which fell between that of the pure gold nanoparticles, and controlled by the mass fraction of the constituent components. If independent transitions were occurring, two separate transitions would be expected, and the particle with a lower LCST would not see a shift in its aggregation. This is also the first example of co-operative aggregation of nanoparticles with different coatings and implies that their responsive transitions involve interaction between the different sized nanoparticles rather than isolated events. As a control experiment, the same PNIPAMs were first mixed in the same weight ratios as above (25 : 75, 50 : 50 and 75 : 25) and then added to the surface gold particles following the same procedure as described above. Whilst the homopolymer coated gold nanoparticles have sharp transition temperature of 55 °C and 74 °C, respectively. The CP of each particle conjugated with pre-mixed polymer lying between that of the two pure polymer coated nanoparticles are shown in Fig. 5B and in agreement with what was seen in the single component nanoparticles mixtures. Taken together this shows that the transition of nanoparticles is highly dependant on the whole mixture and that more complex interactions between particles of different coatings take place.

**Fig. 6:**
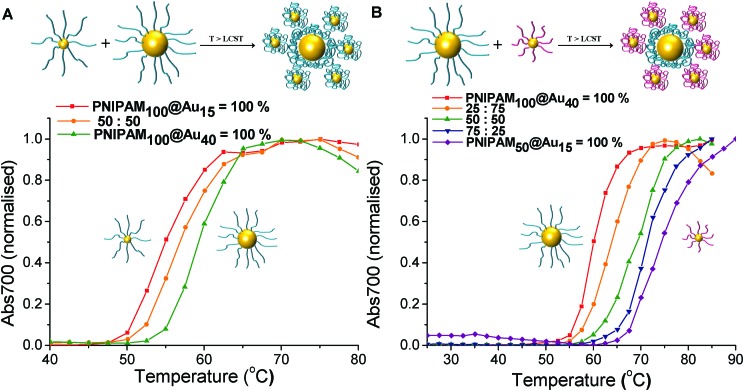
Turbidimetry scans (absorbance at 700 nm) of (A) blends with different mass fraction of PNIPAM_100_ coated Au_15_ and Au_40_, (B) PNIPAM_50_@Au_15_ and PNIPAM_100_@Au_40_ mixture with different mass fraction in PBS solution. In all cases the total gold core concentration of the solutions was 0.029 mg mL^–1^.

The above data confirms that co-operativity occurs, in that the observed transitions temperatures are shifted. For this hypothesis to be true, it must be that the lower LCST particles preferentially interact (or aggregate) with the higher LCST ones, or else the transition would not be affected. To enable us to probe and visualise this directly, a different co-operative system was required which enables discrimination between particles. Therefore mixtures of differently sized nanoparticles were chosen, as this would give a measurable about in the TEM imaging (*vide infra*). As shown in Fig. 6A, with the same polymer PNIPAM_100_ coating, transition of 15 nm gold nanoparticles occurs at 55 °C, while 40 nm gold nanoparticles shifted the transition to 60 °C. The solution containing a 50 : 50% mixture of the particles shows a single transition at 57 °C. Fig. 6B shows turbidimetry scans for blends of different particle with the different polymer coating. The PNIPAM_100_ coated 40 nm gold nanoparticle had an LCST of 60 °C, whereas the PNIPAM_50_ coated 15 nm gold nanoparticle had an LCST of 74 °C. LCSTs of particle mixture for 25 : 75%, 50 : 50% and 75 : 25% with relative weight percent of PNIPAM_50_ coated 15 nm gold nanoparticles and PNIPAM_100_ coated 40 nm gold nanoparticles were measured. In all cases, a single transition of particle mixture was observed 64 °C, 69 °C and 72 °C, showing that LCST is controlled by the relative weight fraction of each particles. This result proved process successfully and the LCST of mixture particle solution is determined as a consequence of relative weight proportion of individual polymer coated nanoparticles. This strategy can be useful to control and predict LCSTs of polymer coated nanoparticles for desired condition.

**Fig. 7:**
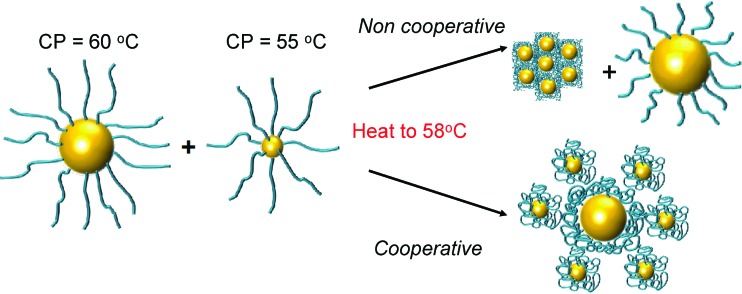
Schematic to show co-operative and non-cooperative aggregates expected to guide TEM analysis (below).

**Fig. 8:**
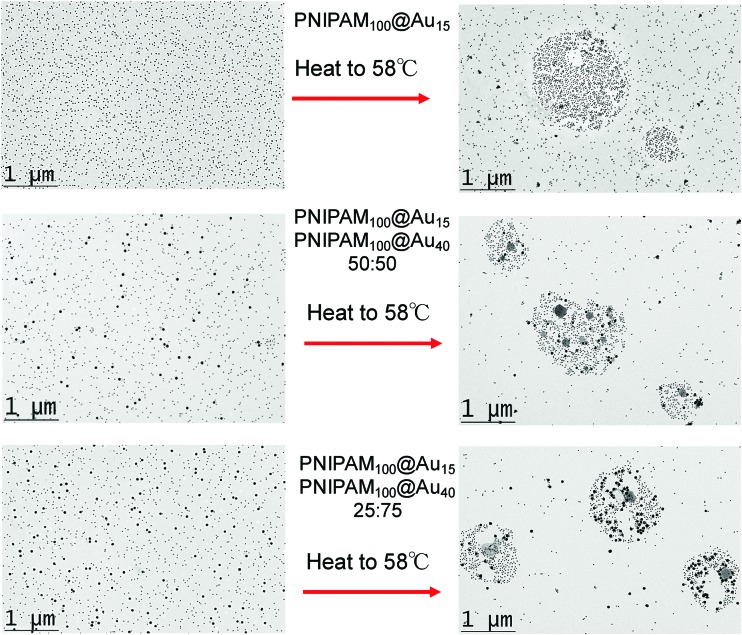
TEM analysis of co-operative particle aggregation. Left hand column shows nanoparticles at 25 °C (below their cloud point) and right hand column shows the same particles which were prepared at 58 °C, above cloud point of the 15 nm particles, but below that of the 40 nm particles.

The above data shows the highly co-operative nature of the nanoparticle transitions, but to it is desirable to be able to quantify the co-operativity and provide direct evidence that different nanoparticles are actually interacting preferentially with their partners. Fig. 7 shows a scheme of the possible outcomes which would be expected for co-operative and non-cooperative transitions with different size/CP particles. In the non-cooperative case, heating above the CP of the smaller particle (lower CP) would be expected to only lead to aggregates of small particles forming, as the larger (higher CP) particles would still be well dispersed. Conversely, if co-operative aggregation was occurring, then mixed aggregates containing both particles should be formed, explaining the modulation. To probe this effect a range of nanoparticle samples (both pure and mixed) were prepared at a range of temperatures both below the CP but also at the CP. By choosing at temperature at the CP the larger gold nanoparticles (with high CPs) if they are not interacting should not aggregate at this temperature, providing a read out for the co-operativitiy. A range of mixtures were prepared of PNIPAM_100_@Au_15_ : PNIPAM_100_@Au_40_ with weight ratios of 3 : 1, 1 : 1 and 1 : 3 at both 25 °C and 58 °C (*i.e.* below and at CP) and the TEM images are shown in Fig. 8 and Fig. S8 in the ESI.†

Fig. 8 revealed that in the mixed nanoparticle solutions, mixed aggregates are forming containing both small and large particles, rather than just those of the lower CP material, which would be expected in a non-cooperative environment as the temperature was below the aggregation temperature of the high CP material. It should be noted that particles generated with other polymer coatings, or with higher/lower grafting densities may not show the same behaviour, as we have reviewed in detail previously.^31^ For instance, the grafting to approach may provide sufficiently spaced polymers that inter-rather than intra particle interactions dominate and hence leading to the observations made here.

In addition to be a useful method for fine-tuning transition temperatures this shows that the particles aggregation is clearly dependant on the other particles which they are in contact with and may offer opportunities particularly in biosensing where the aggregation of a small fraction of a sample could be enhanced by other, non-interacting fractions of particles. This approach also shows that post-synthesis, obtaining an incorrect (or non-desired) transition temperature can be overcome though simple mixing.

## Conclusions

Here we have investigated the co-operative aggregation of a range of thermo-responsive polymer coated gold nanoparticles. It was shown that these particles were tolerant to almost all possible mixing parameters to still produce a single transition; (i) different polymers on same sized particles, (ii) mixture of polymers on same sized particles, (iii) same polymer on different sized particles and (iv) different polymers on different sized particles in all cases enabling a route to fine-tune the transition temperature. Crucially, using the co-operative aggregation of differently sized nanoparticles it was possible to obtain evidence for the interaction of large/small nanoparticles with different cloud points *via* the formation of mixed aggregations in TEM, rather than the single-sized aggregates. Such observations not only provide insight in the mechanism of these aggregation but provide an opportunity to create nanomaterials which are capable of interacting with their environment, and also communicating this to other particles in the mixture, which could offer new opportunities in biosensing.

## Experimental section

### Materials

All chemicals were used as supplied unless otherwise stated. Methanol, hexane, hydrochloric acid, dichloromethane, toluene, acetone, tetrahydrofuran and diethyl ether were purchased from Fisher Scientific at laboratory reagent grade unless otherwise stated. Deuterochloroform (99.9 atom% D), 4,4′-azobis(4-cyanovaleric acid) (>97.0%), dodecane thiol (≥98.0%), potassium phosphate tribasic (reagent grade, ≥98.0%), carbon disulfide (≥99.9%), 2-bromo-2-methylpropionic acid (98.0%), *N*-isopropylacrylamide (97.0%), mesitylene (analytical standard) and magnesium sulfate (≥99.5%) were all purchased from Sigma-Aldrich. Ultrahigh quality water with a resistance of 18.2 MΩ cm (at 25 °C) was obtained from a Millipore Milli-Q gradient machine fitted with a 0.22 μm filter. Gold nanoparticle solutions for 15 nm (0.242 mmol L^–1^) and 40 nm (0.296 mmol L^–1^) were purchased from BBI Solutions. Pre-formulated, powdered, phosphate buffered saline was purchased from Sigma-Aldrich, and the desired solution made by addition of ultrahigh quality water to give [NaCl] = 0.137 M, [KCl] = 0.0027 M, [Na_2_HPO_4_] = 0.01 M, [KH_2_PO_4_] = 0.0018 M and pH = 7.4.

### Analytical and physical methods

^1^H and ^13^C NMR spectra were recorded for analysis of monomer conversions and polymer compositions on Bruker DPX-400 spectrometer using deuterated solvents obtained from Sigma-Aldrich. All chemical shifts are reported in ppm (*δ*) relative to tetramethylsilane (TMS). FTIR spectra were acquired using a Bruker Vector 22 FTIR spectrometer with a Golden Gate diamond attenuated total reflection cell. A total 64 (or 128) scans with resolution of 4 cm^–1^ were collected. Samples were pre-dried as a thin film for FTIR analysis. SEC analysis was conducted on Varian 390-LC MDS system equipped with a column, two PL-AS RT/MT auto sampler, a PL-gel 3 mm (50 × 7.5 mm) guard column, two PL-gel 5 mm (300 × 7.5 mm) mixed-D columns using dimethylformamide (DMF) with 1 mg mL^–1^ LiBr at 50 °C as the eluent at a flow rate of 1.0 mL min^–1^. The GPC system was equipped with ultraviolet (UV) (set at 280 nm) and differential refractive index (DRI) detections. Narrow molecular weight poly(methyl methacrylate) (PMMA) standards (200–1.0 × 106 g mol^–1^) were used for calibration using a second order polynomial fit. Polymer solutions at 1 mg mL^–1^ were prepared in the eluent and filtered through 0.45 mm filters prior to injection. UV-vis spectra were recorded in a disposable cuvette using a Cary 60 UV-vis spectrometer from Agilent at 25 °C. Lower critical solution temperatures of free PNIPAM and PNIPAM nanoparticles were also analyzed using a Agilent Cary 60 UV-vis spectrometer equipped with a temperature controller at 700 nm with a heating/cooling rate of 1 °C min^–1^. The cloud point of PNIPAM and PNIPAM nanoparticles were determined by normalising the turbidimetry curve such that the values were in the range of 0 to 1, and the transition temperature was defined as being the temperature corresponding to a normalised absorbance of 0.5. A polymer concentration of 2.5 mg mL^–1^ was used in all experiments. DLS and zeta potential measurements were performed using a Nano-Zs from Malvern Instruments, UK running DTS software (4 mW, He–Ne laser, *λ* = 633 nm) and an avalanche photodiode (APD) detector. The scattered light was measured at an angle of 173° for DLS measurement and at 12.8° for zeta potential measurements. The temperature was stabilized to ±0.1 °C of the set temperature. All samples were prepared at the concentration of 0.029 mg mL^–1^ gold nanoparticles. Hydrodynamic radii and zeta potential were determined using the manufacturer's software. The size and morphology of the synthesized gold nanoparticles and polymer coated gold nanoparticles were estimated by JEOL 2000FX transmission electron microscopy (TEM) at an accelerating voltage 200 kV. A drop of sample solution was deposited onto a copper grid and the water was evaporated under air. No staining was applied. The X-ray photoemission spectroscopy (XPS) data were collected at the Warwick Photoemission Facility, University of Warwick, more details of which are available at reference.^32^ The samples investigated in this study were deposited on to Cu foil, mounted on to a sample bar and loaded in to a Kratos Axis Ultra DLD spectrometer which possesses a base pressure of ∼5 × 10^–10^ mbar. XPS measurements were performed in the main analysis chamber, with the sample being illuminated using an Al Kα X-ray source. The measurements were conducted at room temperature and at a take-off angle of (30°) with respect to the surface parallel. The core level spectra were recorded using a pass energy of 20 eV (resolution approx. 0.4 eV). The spectrometer work function and binding energy scale were calibrated using the Fermi edge and 3d_5/2_ peak recorded from a polycrystalline Ag sample immediately prior to the commencement of the experiments. The data were analysed in the CasaXPS package, using Shirley backgrounds, mixed Gaussian–Lorentzian (Voigt) lineshapes. For compositional analysis, the analyser transmission function has been determined using Ag, Au and Cu foils to determine the detection efficiency across the full binding energy range.

### Synthesis of 2-(dodecylthiocarbonothioylthio)-2-methylpropanoic acid (DMP)

Dodecane thiol (4.00 g, 4.73 mL, 19.76 mmol) was added dropwise to a stirred suspension of K_3_PO_4_ (4.20 g, 19.76 mmol) in acetone (60 mL) over 25 minutes. CS_2_ (4.10 g, 3.24 mL, 53.85 mmol) was added and the solution turned bright yellow. After stirring for ten minutes 2-bromo-2-methylpropionic acid (3.00 g, 17.96 mmol) was added and a precipitation of KBr was noted. After stirring for 16 hour, the solvent was removed under reduced pressure and the residue was extracted into CH_2_Cl_2_ (2 × 200 mL) from 1 M HCl (200 mL). The organic extracts were washed with water (200 mL) and brine (200 mL) and further dried over MgSO_4_. The solvent was removed under reduced pressure and the residue was purified by recrystallization in hexane.

### Polymerisation of *N*-isopropylacrylamide using 2-(dodecylthiocarbonothioylthio)-2-methylpropanoic acid (DMP)

Polymers with three different molecular weights were synthesised in typical procedure.^33^ *N*-Isopropylacrylamide (1 g, 8.84 mmol), 2-(dodecylthiocarbonothioylthio)-2-methylpropanoic acid (32.22 mg, 88.4 μmol), and 4,4′-azobis(4-cyanovaleric acid) (ACVA) (4.95 mg, 17.7 μmol) were dissolved in methanol/toluene (1 : 1; 4 mL) in a glass vial containing a stir bar giving [monomer] : [chain transfer agent] : [initiator] = 100 : 1 : 0.2. Mesitylene (150 μL) was added as an internal reference and the mixture was stirred (5 min). An aliquot of this starting mixture was removed for ^1^H NMR analysis. The vial was fitted with a rubber septum and degassed by bubbling with nitrogen gas (30 min). The vial was then placed in an oil bath thermostated at 70 °C. After 35 minutes, the reaction mixture was opened to air and quenched in liquid nitrogen. An aliquot was removed and conversion determined by ^1^H NMR. The remainder was precipitated into diethyl ether (45 mL). The polymer was re-precipitated and purified from THF to diethyl ether three times. The product was purified three times by precipitation from toluene into diethyl ether, isolated centrifugation, and dried under vacuum overnight to give a yellow solid. The overall monomer conversion was determined from the ^1^H NMR spectrum by measuring the decrease in intensity of the vinyl peaks associated with the monomer relative to mesitylene. Conversion (NMR): 72%; *M*_n_ (theoretical), 8100 g mol^–1^; *M*_n_ (SEC), 7700 g mol^–1^; *M*_w_/*M*_n_ (SEC), 1.10.

### General procedure for the synthesis of polymer-coated gold nanoparticles

Approximately 1 mg of the desired thiol-terminated polymer was added to a microcentrifuge tube, and dissolved in 100 μL of high-purity water. To this tube was added 900 μL of the citrated-stabilized gold nanoparticle solution (15 nm: 0.242 mmol L^–1^, 40 nm: 0.296 mmol L^–1^ total gold concentration), which was then agitated overnight in the absence of light. To remove excess polymer, the particles were centrifuged for 30 minutes at 10 000 rpm. Following careful decantation of the supernatant, the particles were then re-dispersed in 1 mL of high-quality water and the centrifugation–resuspension process repeated for a total of 3 cycles. After the final cycle the particles were dispersed in 1 mL of high-quality water for future use. Assuming complete incorporation of the citrate coated gold particles into the final polymer coated particles the total concentration of gold in the final solution was 0.242 mmol L^–1^, 0.048 mg mL^–1^ and 0.296 mmol L^–1^, 0.058 mg mL^–1^.
