# Supplementary material for: Co-operative transitions of responsive-polymer coated gold nanoparticles; precision tuning and direct evidence for co-operative aggregation
Source: J Mater Chem B. 2016 Aug 2;4(34):5673–82. doi: 10.1039/c6tb01336h (PMC5038384; doi:10.1039/c6tb01336h)
Supplement: Supplementary file 1 [file TB-004-C6TB01336H-s001.pdf]

Electronic Supplementary Material (ESI) for Journal of Materials Chemistry B.
This journal is © The Royal Society of Chemistry 2016

Supporting Information for;

Co-operative Transitions of Responsive-Polymer Coated Gold Nanoparticles; Precision Tuning and Direct Evidence for Co-operative Aggregation

Sangho Won[a], Daniel J. Phillips[a], Marc Walker[b] and Matthew I. Gibson[a,c]*

a) Department of Chemistry, University of Warwick, Coventry, CV4 7AL, United Kingdom

b) Department of Physics, University of Warwick, Coventry, CV4 7AL, United Kingdom

c) Warwick Medical School, University of Warwick, Coventry, CV4 7AL, United Kingdom

Corresponding Author E-mail: M.I.Gibson@warwick.ac.uk

Additional Characterisation Data

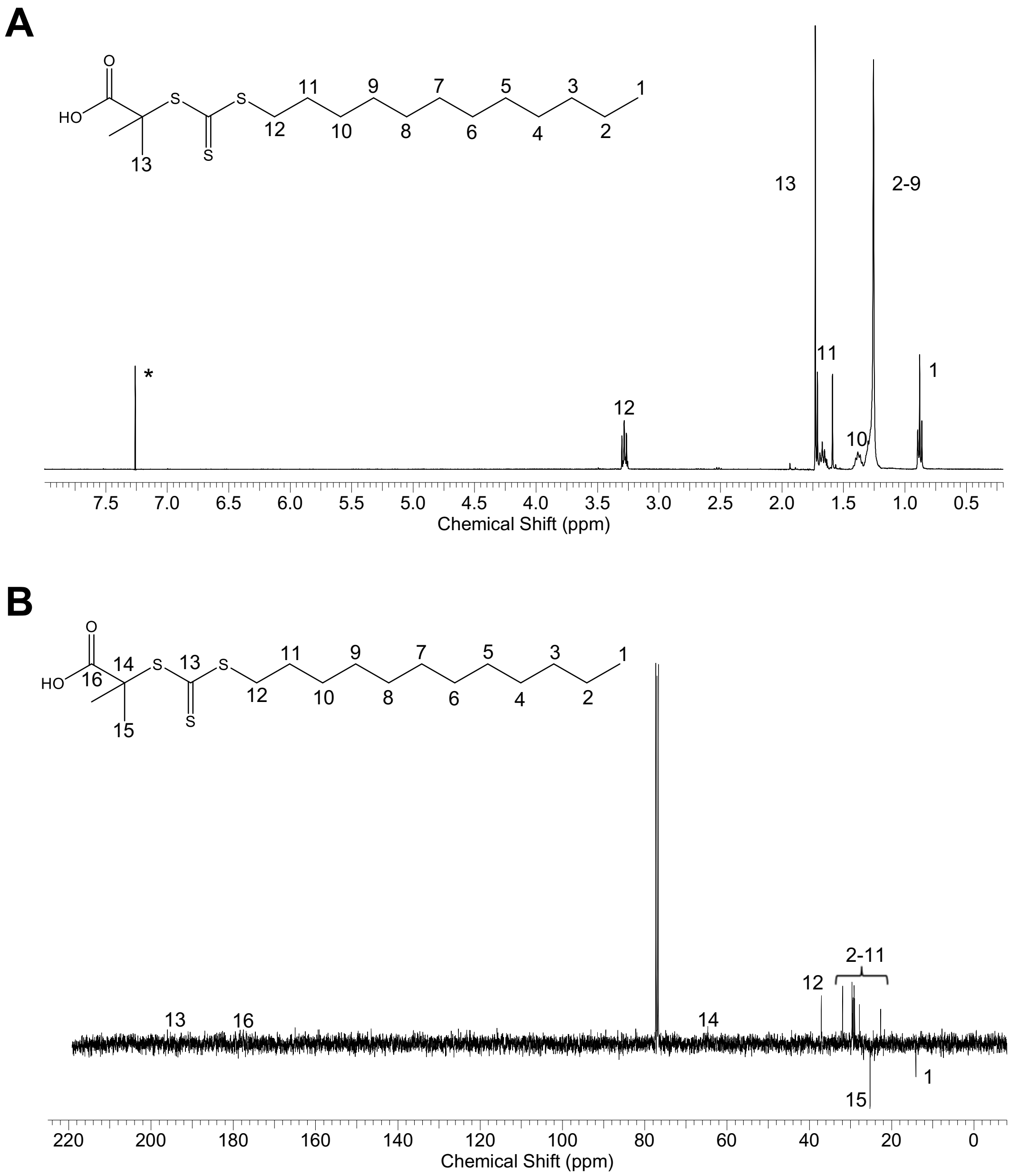

Figure S1. ^{1}H NMR and ^{13}C NMR spectra of 2-(dodecylthiocarbonothioylthio)-2-methylpropanoic acid in $CDCl_3$. *=solvent

^{1}H NMR (400 MHz, $CDCl_3$) δ_{ppm}: 3.28 (2H, t, $J_{12\text{-}11}$= 7.54 Hz, H^{12}); 1.71 (6H, s, H^{13}); 1.67 (2H, p, $J_{11\text{-}10,\ 11\text{-}12}$ = 7.53 Hz, H^{11}); 1.38 (2H, p, $J_{10\text{-}9,\ 10\text{-}11}$= 7.78 Hz, H^{10}); 1.26 (16H, m, $H^{2\text{-}9}$); 0.88 (3H, t, $J_{1\text{-}2}$= 7.03 Hz, H^{1}).

^{13}C NMR (400 MHz, $CDCl_3$) δ_{ppm}: 196.00 (C^{13}); 177.56 (C^{16}); 64.69 (C^{14}); 37.09 (C^{12}); 31.92, 29.71, 29.63, 29.56, 29.45, 29.35, 29.12, 22.70 (C^{2-9}); 28.97 (C^{10}); 27.81 (C^{11}); 25.25 (C^{15}); 14.13 (C^{1}).

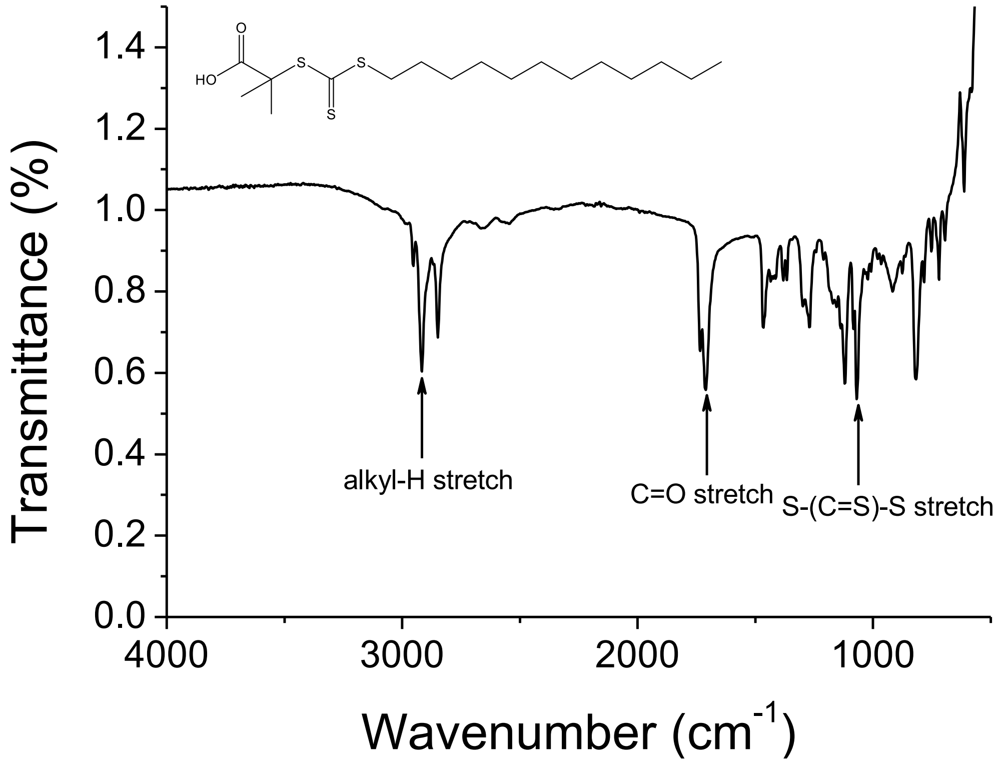

Figure S2. Infrared spectra of 2-(dodecylthiocarbonothioylthio)-2-methylpropanoic acid.

IR cm^{-1}: 2917 (alkyl-H stretch); 1712 (C=O stretch); 1070(S-(C=S)-S stretch).

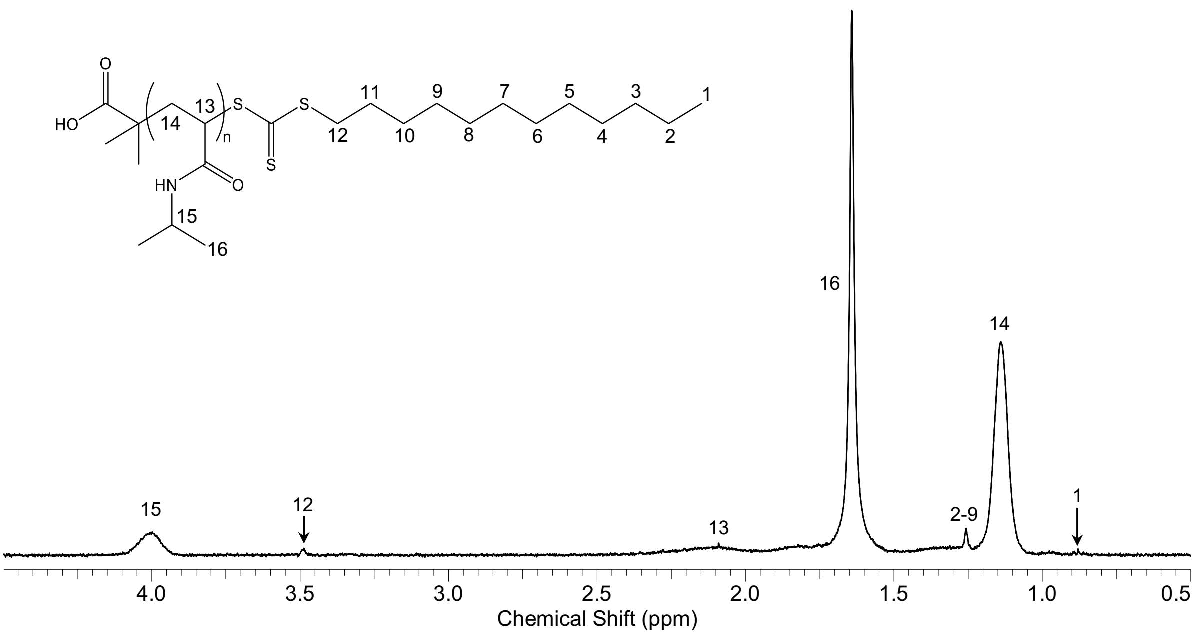

Figure S3. ^{1}H NMR spectra of poly(N-isopropylacrylamide).

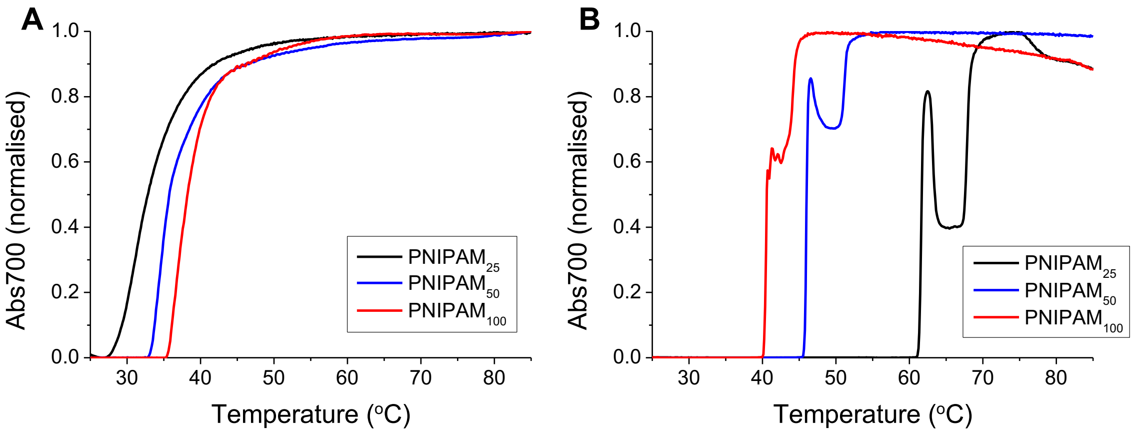

Figure S4. Turbidimetry scans (absorbance at 700 nm) of homopolymer in a) pure water and b) PBS. In all cases the total polymer concentration of the solutions was 2.5 mg mL^{-1}.

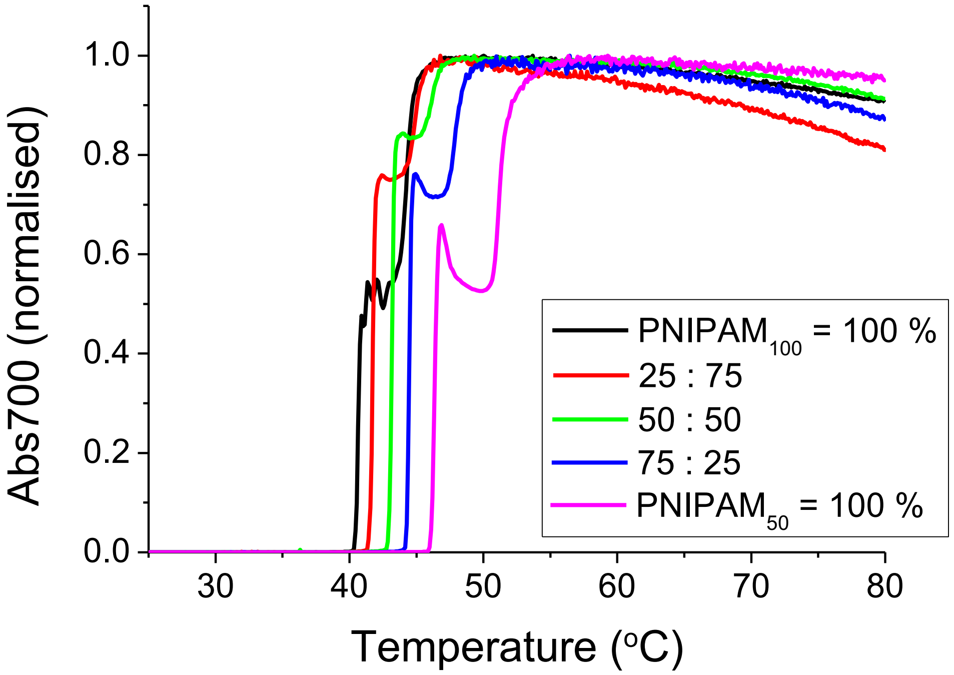

Figure S5. Turbidimetry analysis of blends of $PNIPAM_{50}$ and $PNIPAM_{100}$ at 2.5 mg mL^{-1}.

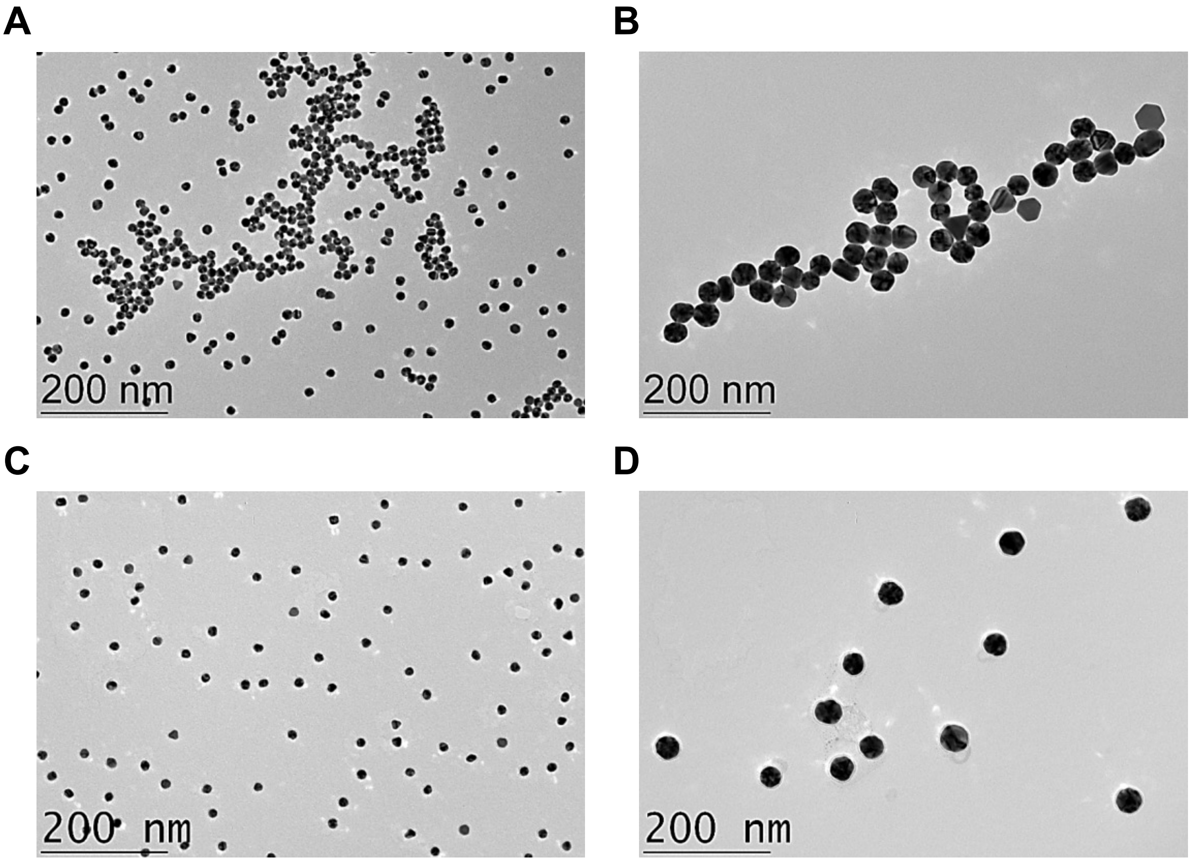

Figure S6. TEM images of uncoated a) 15nm and b) 40nm sized bare gold nanoparticles and $PNIPAM_{100}$ coated c) 15nm and d) 40nm sized gold nanoparticles (higher colloidal stability).

Table S1. XPS elemental ratios for PNIPAM/gold hybrid nanoparticles.

Sample	Au 4f [%]	C 1s [%]	O 1s [%]	N 1s [%]	Cu 2p [%]	S 2p [%]
**Bare Au_{15}**	2.54	45.24	41.33	0.07	10.83	-
**$PNIPAM_{100}$@Au_{15}**	1.66	75.38	17.79	3.80	1.36	0.02
**$PNIPAM_{50}$@Au_{15}**	2.6	70.35	19.83	4.83	2.36	0.02
**$PNIPAM_{25}$@Au_{15}**	3.53	70.68	18	4.79	2.99	0.01
**Cu foil reference**	-	-	-	59.94	40.06	-

Sample	Au 4f [%]	C 1s [%]	O 1s [%]	N 1s [%]	Cu 2p [%]	S 2p [%]
**Bare Au_{40}**	1.00	17.81	16.27	0.03	4.26	-
**$PNIPAM_{100}$@Au_{40}**	1.00	45.41	10.72	2.29	0.82	0.012
**$PNIPAM_{50}$@Au_{40}**	1.00	27.06	7.63	1.86	0.91	0.008
**$PNIPAM_{25}$@Au_{40}**	1.00	20.02	5.10	1.36	0.85	0.003
**Cu foil reference**	-	-	-	59.94	40.06	-

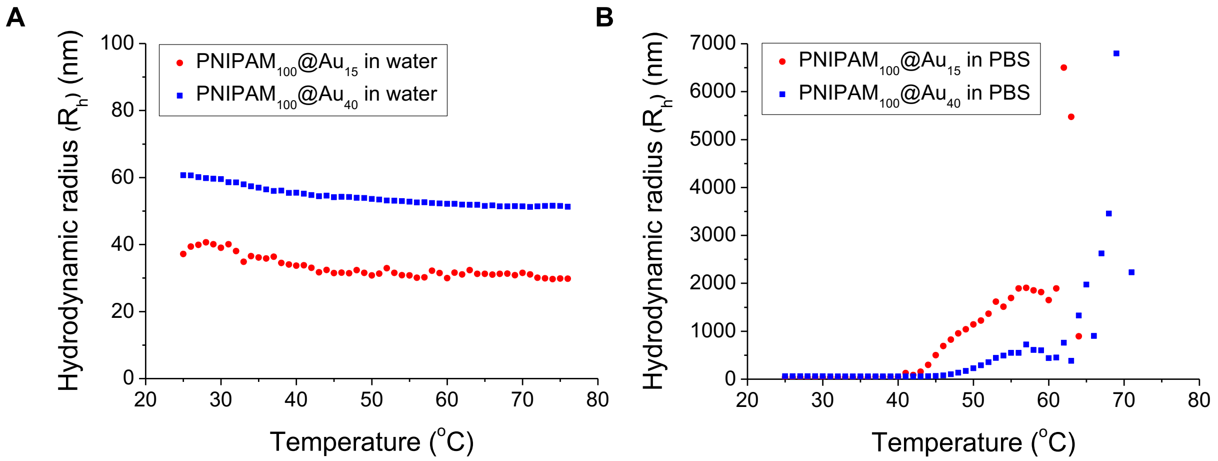

Figure S7. Hydrodynamic radius (Rh) for the PNIPAM coated 15 nm and 40 nm gold nanoparticles in a) pure water and b) PBS buffer as a function of temperature.

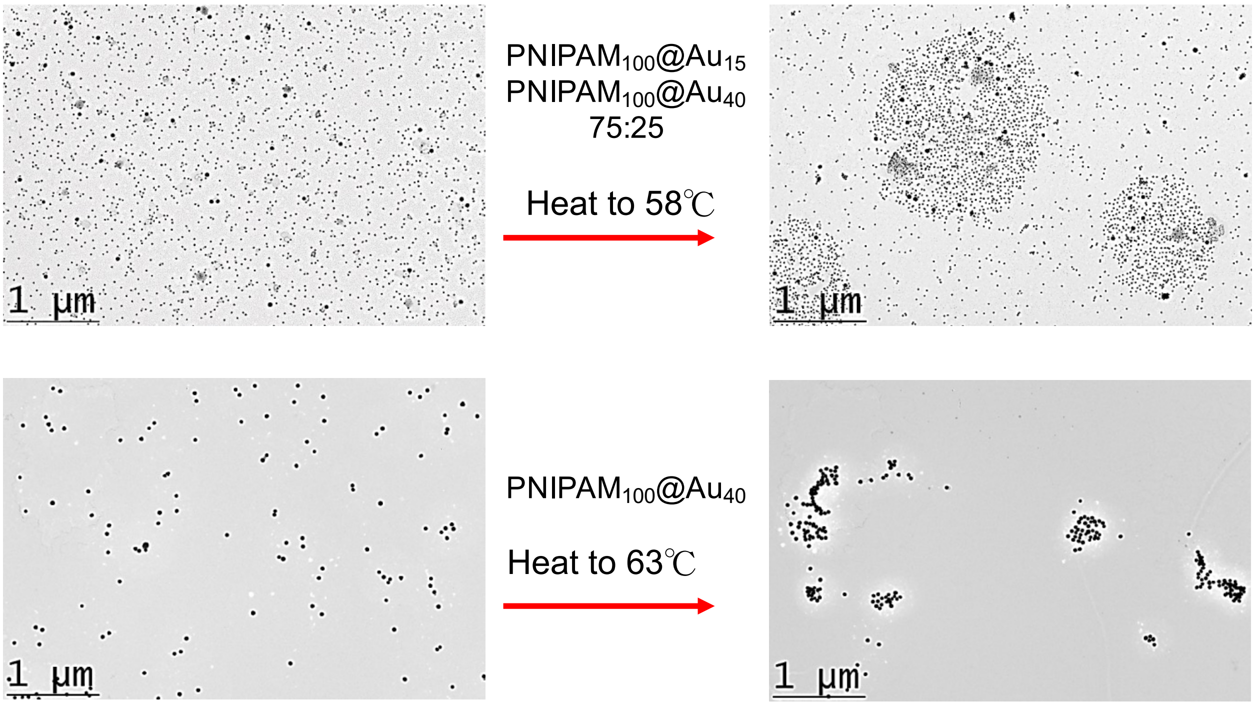

Figure S8. Additional TEM analysis of co-operative particle aggregation. Left hand column shows nanoparticles at 25 °C (below their cloud point). Right hand column shows the same particles which were prepared above cloud point of the 15 nm particles (58 °C) and 40 nm particles (63 °C).
